# 6-Chloro-2-chloro­methyl-4-phenyl­quinazoline 3-oxide

**DOI:** 10.1107/S1600536814005303

**Published:** 2014-03-15

**Authors:** Thammarse S. Yamuna, Jerry P. Jasinski, Manpreet Kaur, Hemmige S. Yathirajan, Maravanahalli S. Siddegowda

**Affiliations:** a Department of Studies in Chemistry, University of Mysore, Manasagangotri, Mysore 570 006, India; b Department of Chemistry, Keene State College, 229 Main Street, Keene, NH 03435-2001, USA; c R. L. Fine Chem, Bengaluru, 560 064, India

## Abstract

In the title compound, C_15_H_10_Cl_2_N_2_O, the dihedral angle between the mean planes of the phenyl ring and the 10-membered quinazoline ring is 63.3 (4)°. In the crystal, pairs of weak C—H⋯O inter­actions link the mol­ecules into centrosymmetric dimers, forming *R*_2_^2^(10) graph-set ring motifs. In addition, weak π–π stacking inter­actions [minimum centroid–centroid separation = 3.6810 (8) Å] are observed, which contribute to the formation of a supramolecular assembly in the packing array.

## Related literature   

For general background and the pharmacological properties of quinazoline derivatives, see: Andries *et al.* (2005 ▶); Al-Rashood *et al.* (2006 ▶); Ghorab *et al.* (2010*a* ▶,*b* ▶,*c* ▶); Harris & Thorarensen (2004 ▶); Jantova *et al.* (2004 ▶); Rádl *et al.* (2000 ▶); Klepser & Klepser (1997 ▶). For related structures, see: Brown & Gainsford (1979 ▶); El-Brollosy *et al.* (2012 ▶); Shi *et al.* (2004 ▶); Suguna *et al.* (1982 ▶); Xie & Li (2006 ▶). For standard bond lengths, see: Allen *et al.* (1987 ▶).
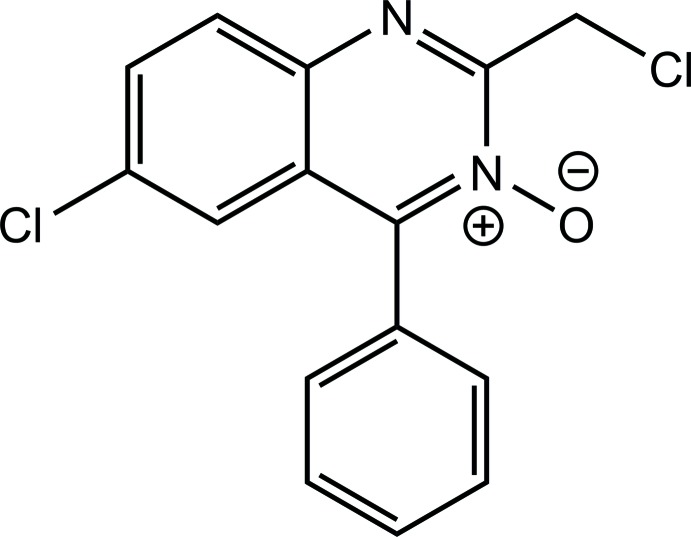


## Experimental   

### 

#### Crystal data  

C_15_H_10_Cl_2_N_2_O
*M* *_r_* = 305.15
Monoclinic, 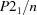

*a* = 8.2030 (3) Å
*b* = 14.3203 (5) Å
*c* = 11.8477 (4) Å
β = 105.016 (4)°
*V* = 1344.22 (9) Å^3^
*Z* = 4
Mo *K*α radiation
μ = 0.48 mm^−1^
*T* = 173 K
0.22 × 0.16 × 0.08 mm

#### Data collection  

Agilent Xcalibur (Eos, Gemini) diffractometer
Absorption correction: multi-scan (*CrysAlis PRO* and *CrysAlis RED*; Agilent, 2012 ▶) *T*_min_ = 0.829, *T*_max_ = 1.000
17250 measured reflections
4599 independent reflections
3778 reflections with *I* > 2σ(*I*)
*R*_int_ = 0.033

#### Refinement  

*R*[*F* ^2^ > 2σ(*F* ^2^)] = 0.038
*wR*(*F* ^2^) = 0.100
*S* = 1.03
4599 reflections
181 parameters
H-atom parameters constrained
Δρ_max_ = 0.44 e Å^−3^
Δρ_min_ = −0.48 e Å^−3^

### 

Data collection: *CrysAlis PRO* (Agilent, 2012 ▶); cell refinement: *CrysAlis PRO*; data reduction: *CrysAlis PRO*; program(s) used to solve structure: *SUPERFLIP* (Palatinus & Chapuis, 2007 ▶); program(s) used to refine structure: *SHELXL2012* (Sheldrick, 2008 ▶); molecular graphics: *OLEX2* (Dolomanov *et al.*, 2009 ▶); software used to prepare material for publication: *OLEX2*.

## Supplementary Material

Crystal structure: contains datablock(s) I. DOI: 10.1107/S1600536814005303/zs2289sup1.cif

Structure factors: contains datablock(s) I. DOI: 10.1107/S1600536814005303/zs2289Isup2.hkl

Supporting information file. DOI: 10.1107/S1600536814005303/zs2289Isup3.cml

CCDC reference: 990666

Additional supporting information:  crystallographic information; 3D view; checkCIF report

## Figures and Tables

**Table 1:** Hydrogen-bond geometry (Å, °)

*D*—H⋯*A*	*D*—H	H⋯*A*	*D*⋯*A*	*D*—H⋯*A*
C15—H15*A*⋯O1^i^	0.97	2.57	3.4199 (16)	146

Symmetry code: (i) 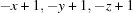.

**Fig. 1.**
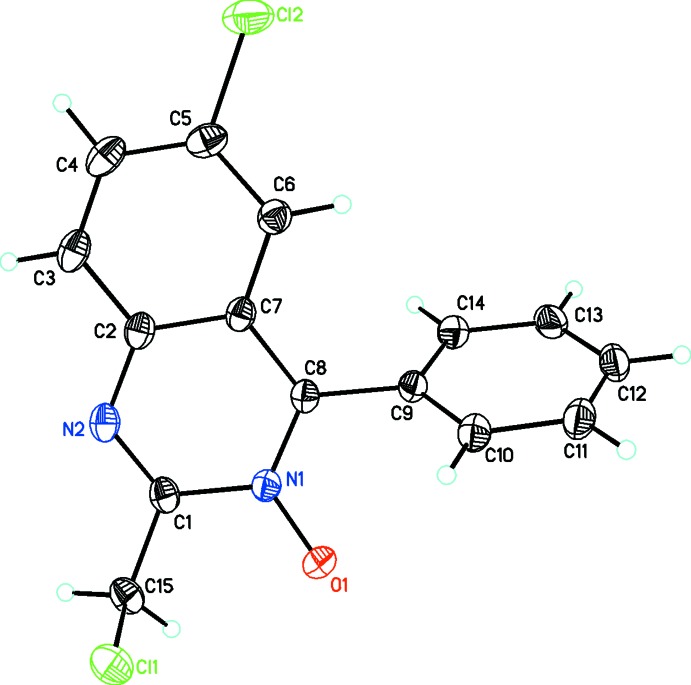
ORTEP drawing of (I) (C15H11Cl2N2O) showing the labeling scheme with 30% probability displacement ellipsoids.

**Fig. 2.**
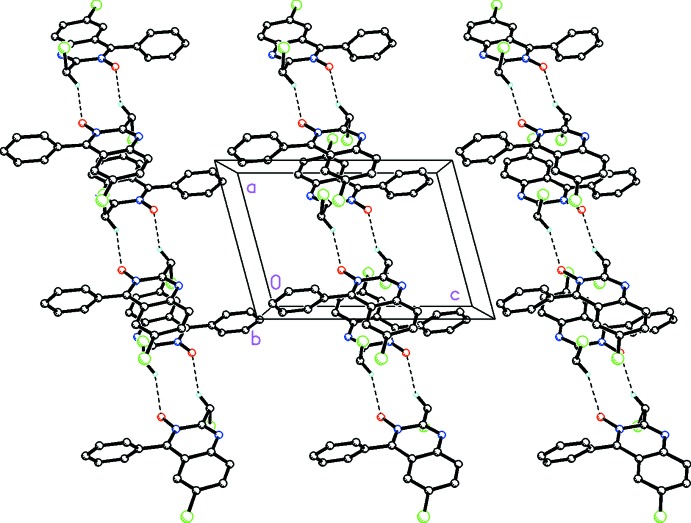
Molecular packing for (I) viewed along the b axis. Dashed lines indicate weak C—H···O intermolecular interactions. H atoms not involved in hydrogen bonding have been removed for clarity.

**Fig. 3.**
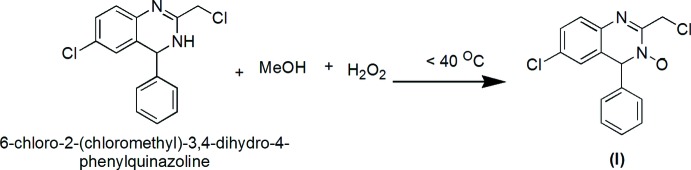
: Synthesis scheme of (I).

## supplementary crystallographic information

### 1. Comment

Quinazolines have been intensively studied for their interesting pharmacological properties such as anticancer activity (Ghorab *et al.*, 2010**a*,*b*,c*). A number of quinozolines have also been clinically used as antifungal, antibacterial and antiprotozoic drugs (Jantova *et al.*, 2004; Harris & Thorarensen, 2004) and antituberculotic agents (Andries *et al.*, 2005) and have pharmacological properties which include antitumor (Al-Rashood *et al.*, 2006) and analgesic (Rádl *et al.*, 2000) properties. Dihydropyrimidine derivatives (DHPMs) may also be applied as antimicrobial, anti-inflammatory and quinazoline analogs and have showed remarkable activity against the opportunistic infections of some microorganisms proved to be the prinicipal cause of death in patients with immunocompromised diseases such as acquired immune deficiency syndrome (Klepser & Klepser, 1997) and fused quinazoline systems, which are also important pharmacophores. The crystal structures of some related compounds, viz., 2-phenylquinazoline 1,3-dioxide (Brown & Gainsford, 1979), 1-{[(2,3-dihydro-1*H*-inden-2-yl)oxy]methyl}quinazoline-2,4(1*H*, 3*H*)-dione (El-Brollosy *et al.*, 2012), 3-(4-chlorophenyl)-3,4-dihydroquinazolin-2(1*H*)-one (Shi *et al.*, 2004), (4*S*)-2,4-dimethyl-1,2-dihydropyrazino[2,1-b]quinazoline-3(4*H*)- 6-dione (Suguna *et al.*, 1982) and 2-diethylamino-3-phenylquinazolin- 4(3*H*)-one (Xie *et al.*, 2006), have been reported. In view of the importance of the title compound, (I), C_15_H_11_Cl_2_N_2_O, this paper reports its crystal structure.

In the title compound the dihedral angle between the mean planes of the phenyl ring and the 10-membered quinazolin ring is 63.3 (4)° (Fig. 1). Bond lengths are in normal ranges (Allen *et al.*, 1987). In the crystal, a weak C15—H15*A*···O1 intermolecular interaction link the molecules into centrosymmetric dimers forming *R*_2_^2^(10) graph set ring motifs (Fig. 2). In addition, weak Cg1—Cg3 and Cg2—Cg3 π—π stacking intermolecular interactions are observed which contribute to crystal packing stability (Cg1—Cg3 = 3.6810 (8)Å; *x* - 1/2,-*y* + 1/2, *z* - 1/2; Cg2—Cg3 = 3.8821 (8)Å; *x* + 1/2,-*y* + 1/2, *z* - 1/2; Cg1 = N(1)/C(1)/N(2)/C(2)/C(7)/C(8); Cg2 = C2–C7; Cg3 = C9–C14). No classical hydrogen bonds were found.

### 2. Experimental

6-chloro-2-(chloromethyl)-3,4-dihydro-4-phenylquinazoline (10 g, 0.03434 mol) was dissolved in 40 ml of methanol and stirred for 5 mins at room temperature. To this mixture, 10 g of 50% H_2_O_2_ solution (5 g, 0.147 mol) was added dropwise over 30 mins, maintaining the temperature below 313 K, then stirred for 6 hrs in a RB flask, cooled, filtered and dried at 333 K (Fig. 3). The precipitate was dissolved in a (1:1) mixture of toluene and methylene dichloride at 313 K. After a few days, X-ray quality crystals appeared on slow evaporation.

### 3. Refinement

All of the H atoms were placed in their calculated positions and then refined using the riding model with C—H bond lengths of 0.93Å (CH) or 0.97Å (CH_2_). Isotropic displacement parameters for these atoms were set to 1.2 (CH, CH_2_) times *U*_eq_ of the parent atom.

### Crystal data

**Table:**

C_15_H_10_Cl_2_N_2_O	*F*(000) = 624
*M**_r_* = 305.15	*D*_x_ = 1.508 Mg m^−^^3^
Monoclinic, *P*2_1_/*n*	Mo *K*α radiation, λ = 0.71073 Å
*a* = 8.2030 (3) Å	Cell parameters from 5430 reflections
*b* = 14.3203 (5) Å	θ = 3.1–32.8°
*c* = 11.8477 (4) Å	µ = 0.48 mm^−^^1^
β = 105.016 (4)°	*T* = 173 K
*V* = 1344.22 (9) Å^3^	Irregular, colourless
*Z* = 4	0.22 × 0.16 × 0.08 mm

### Data collection

**Table:**

Agilent Xcalibur (Eos, Gemini) diffractometer	4599 independent reflections
Radiation source: Enhance (Mo) X-ray Source	3778 reflections with *I* > 2σ(*I*)
Graphite monochromator	*R*_int_ = 0.033
Detector resolution: 16.0416 pixels mm^-1^	θ_max_ = 32.8°, θ_min_ = 3.1°
ω scans	*h* = −12→12
Absorption correction: multi-scan (*CrysAlis PRO* and *CrysAlis RED*; Agilent, 2012)	*k* = −21→21
*T*_min_ = 0.829, *T*_max_ = 1.000	*l* = −17→17
17250 measured reflections	

### Refinement

**Table:**

Refinement on *F*^2^	Primary atom site location: structure-invariant direct methods
Least-squares matrix: full	Hydrogen site location: inferred from neighbouring sites
*R*[*F*^2^ > 2σ(*F*^2^)] = 0.038	H-atom parameters constrained
*wR*(*F*^2^) = 0.100	*w* = 1/[σ^2^(*F*_o_^2^) + (0.0411*P*)^2^ + 0.6066*P*] where *P* = (*F*_o_^2^ + 2*F*_c_^2^)/3
*S* = 1.03	(Δ/σ)_max_ < 0.001
4599 reflections	Δρ_max_ = 0.44 e Å^−^^3^
181 parameters	Δρ_min_ = −0.48 e Å^−^^3^
0 restraints	

### Special details

**Table:**

Geometry. All esds (except the esd in the dihedral angle between two l.s. planes) are estimated using the full covariance matrix. The cell esds are taken into account individually in the estimation of esds in distances, angles and torsion angles; correlations between esds in cell parameters are only used when they are defined by crystal symmetry. An approximate (isotropic) treatment of cell esds is used for estimating esds involving l.s. planes.

### Fractional atomic coordinates and isotropic or equivalent isotropic displacement parameters (Å^2^)

**Table:**

	*x*	*y*	*z*	*U*_iso_*/*U*_eq_	
Cl1	0.80516 (4)	0.57556 (2)	0.41153 (3)	0.02910 (9)	
Cl2	1.25299 (5)	0.02361 (3)	0.52366 (4)	0.03835 (10)	
O1	0.70795 (12)	0.42471 (7)	0.59735 (8)	0.0258 (2)	
N1	0.77812 (13)	0.37116 (7)	0.53548 (8)	0.01880 (19)	
N2	0.80968 (14)	0.34057 (8)	0.34467 (9)	0.0222 (2)	
C1	0.75384 (16)	0.39185 (9)	0.41604 (10)	0.0206 (2)	
C2	0.90492 (15)	0.26388 (9)	0.38678 (10)	0.0202 (2)	
C3	0.97031 (17)	0.20909 (10)	0.31009 (11)	0.0255 (3)	
H3	0.9429	0.2234	0.2308	0.031*	
C4	1.07367 (17)	0.13503 (10)	0.35109 (12)	0.0269 (3)	
H4	1.1158	0.0984	0.3003	0.032*	
C5	1.11527 (16)	0.11514 (9)	0.47137 (12)	0.0244 (2)	
C6	1.05319 (16)	0.16552 (9)	0.54919 (11)	0.0222 (2)	
H6	1.0832	0.1507	0.6283	0.027*	
C7	0.94247 (15)	0.24056 (8)	0.50668 (10)	0.0188 (2)	
C8	0.86931 (15)	0.29543 (8)	0.58051 (10)	0.0181 (2)	
C9	0.88618 (15)	0.27188 (8)	0.70445 (10)	0.0193 (2)	
C10	0.96680 (16)	0.33233 (9)	0.79356 (10)	0.0229 (2)	
H10	1.0080	0.3895	0.7758	0.027*	
C11	0.98512 (17)	0.30648 (10)	0.90932 (11)	0.0270 (3)	
H11	1.0415	0.3458	0.9693	0.032*	
C12	0.91999 (18)	0.22262 (10)	0.93578 (11)	0.0282 (3)	
H12	0.9316	0.2063	1.0134	0.034*	
C13	0.83771 (18)	0.16286 (10)	0.84755 (12)	0.0273 (3)	
H13	0.7928	0.1069	0.8657	0.033*	
C14	0.82261 (17)	0.18705 (9)	0.73159 (11)	0.0233 (2)	
H14	0.7699	0.1464	0.6720	0.028*	
C15	0.66120 (17)	0.47967 (9)	0.37389 (11)	0.0237 (2)	
H15A	0.5686	0.4876	0.4099	0.028*	
H15B	0.6151	0.4771	0.2898	0.028*	

### Atomic displacement parameters (Å^2^)

**Table:**

	*U*^11^	*U*^22^	*U*^33^	*U*^12^	*U*^13^	*U*^23^
Cl1	0.02893 (17)	0.02459 (16)	0.03348 (17)	−0.00319 (11)	0.00757 (13)	0.00339 (12)
Cl2	0.0404 (2)	0.02622 (17)	0.0529 (2)	0.00878 (14)	0.02027 (18)	0.00153 (15)
O1	0.0310 (5)	0.0258 (5)	0.0227 (4)	0.0072 (4)	0.0109 (4)	−0.0010 (3)
N1	0.0198 (5)	0.0202 (5)	0.0164 (4)	0.0000 (4)	0.0047 (4)	−0.0005 (3)
N2	0.0224 (5)	0.0271 (5)	0.0160 (4)	−0.0036 (4)	0.0032 (4)	−0.0006 (4)
C1	0.0204 (5)	0.0232 (6)	0.0167 (5)	−0.0026 (4)	0.0021 (4)	0.0017 (4)
C2	0.0196 (5)	0.0242 (6)	0.0165 (5)	−0.0044 (4)	0.0041 (4)	−0.0027 (4)
C3	0.0258 (6)	0.0326 (7)	0.0188 (5)	−0.0067 (5)	0.0071 (5)	−0.0074 (5)
C4	0.0255 (6)	0.0295 (6)	0.0283 (6)	−0.0066 (5)	0.0116 (5)	−0.0119 (5)
C5	0.0222 (6)	0.0205 (6)	0.0323 (6)	−0.0023 (4)	0.0102 (5)	−0.0048 (5)
C6	0.0237 (6)	0.0212 (5)	0.0229 (5)	−0.0011 (4)	0.0084 (5)	−0.0002 (4)
C7	0.0194 (5)	0.0205 (5)	0.0170 (5)	−0.0033 (4)	0.0055 (4)	−0.0018 (4)
C8	0.0195 (5)	0.0196 (5)	0.0151 (4)	−0.0020 (4)	0.0044 (4)	−0.0003 (4)
C9	0.0203 (5)	0.0223 (5)	0.0162 (5)	0.0030 (4)	0.0062 (4)	0.0011 (4)
C10	0.0226 (6)	0.0269 (6)	0.0190 (5)	−0.0001 (5)	0.0052 (4)	−0.0005 (4)
C11	0.0247 (6)	0.0378 (7)	0.0176 (5)	0.0051 (5)	0.0042 (5)	−0.0021 (5)
C12	0.0284 (7)	0.0389 (7)	0.0189 (5)	0.0116 (5)	0.0089 (5)	0.0077 (5)
C13	0.0317 (7)	0.0270 (6)	0.0264 (6)	0.0066 (5)	0.0135 (5)	0.0081 (5)
C14	0.0266 (6)	0.0228 (6)	0.0218 (5)	0.0010 (5)	0.0087 (5)	0.0011 (4)
C15	0.0232 (6)	0.0238 (6)	0.0214 (5)	−0.0008 (4)	0.0011 (4)	0.0038 (4)

### Geometric parameters (Å, º)

**Table:**

Cl1—C15	1.7905 (13)	C6—C7	1.4128 (17)
Cl2—C5	1.7366 (14)	C7—C8	1.4194 (16)
O1—N1	1.2943 (13)	C8—C9	1.4778 (15)
N1—C1	1.4086 (15)	C9—C10	1.3926 (17)
N1—C8	1.3467 (15)	C9—C14	1.3921 (17)
N2—C1	1.2901 (16)	C10—H10	0.9300
N2—C2	1.3651 (17)	C10—C11	1.3906 (17)
C1—C15	1.4872 (17)	C11—H11	0.9300
C2—C3	1.4076 (17)	C11—C12	1.383 (2)
C2—C7	1.4134 (16)	C12—H12	0.9300
C3—H3	0.9300	C12—C13	1.384 (2)
C3—C4	1.366 (2)	C13—H13	0.9300
C4—H4	0.9300	C13—C14	1.3909 (17)
C4—C5	1.4058 (19)	C14—H14	0.9300
C5—C6	1.3686 (17)	C15—H15A	0.9700
C6—H6	0.9300	C15—H15B	0.9700
			
O1—N1—C1	118.46 (10)	N1—C8—C9	118.47 (10)
O1—N1—C8	122.39 (10)	C7—C8—C9	122.71 (10)
C8—N1—C1	119.13 (10)	C10—C9—C8	121.01 (11)
C1—N2—C2	119.02 (10)	C14—C9—C8	118.97 (11)
N1—C1—C15	116.24 (11)	C14—C9—C10	120.01 (11)
N2—C1—N1	123.84 (11)	C9—C10—H10	120.3
N2—C1—C15	119.90 (11)	C11—C10—C9	119.40 (12)
N2—C2—C3	119.36 (11)	C11—C10—H10	120.3
N2—C2—C7	120.83 (11)	C10—C11—H11	119.8
C3—C2—C7	119.79 (12)	C12—C11—C10	120.36 (13)
C2—C3—H3	119.7	C12—C11—H11	119.8
C4—C3—C2	120.56 (12)	C11—C12—H12	119.8
C4—C3—H3	119.7	C11—C12—C13	120.47 (12)
C3—C4—H4	120.6	C13—C12—H12	119.8
C3—C4—C5	118.88 (12)	C12—C13—H13	120.2
C5—C4—H4	120.6	C12—C13—C14	119.56 (13)
C4—C5—Cl2	118.63 (10)	C14—C13—H13	120.2
C6—C5—Cl2	118.60 (11)	C9—C14—H14	119.9
C6—C5—C4	122.76 (12)	C13—C14—C9	120.18 (12)
C5—C6—H6	120.7	C13—C14—H14	119.9
C5—C6—C7	118.54 (12)	Cl1—C15—H15A	110.0
C7—C6—H6	120.7	Cl1—C15—H15B	110.0
C2—C7—C8	118.15 (11)	C1—C15—Cl1	108.56 (9)
C6—C7—C2	119.38 (11)	C1—C15—H15A	110.0
C6—C7—C8	122.46 (11)	C1—C15—H15B	110.0
N1—C8—C7	118.80 (10)	H15A—C15—H15B	108.4
			
Cl2—C5—C6—C7	179.38 (9)	C3—C2—C7—C8	−177.77 (11)
O1—N1—C1—N2	−176.01 (11)	C3—C4—C5—Cl2	−177.62 (10)
O1—N1—C1—C15	5.43 (16)	C3—C4—C5—C6	1.7 (2)
O1—N1—C8—C7	−179.81 (11)	C4—C5—C6—C7	0.10 (19)
O1—N1—C8—C9	1.37 (17)	C5—C6—C7—C2	−2.69 (18)
N1—C1—C15—Cl1	80.38 (12)	C5—C6—C7—C8	178.70 (11)
N1—C8—C9—C10	−63.17 (16)	C6—C7—C8—N1	173.70 (11)
N1—C8—C9—C14	117.95 (13)	C6—C7—C8—C9	−7.54 (18)
N2—C1—C15—Cl1	−98.24 (12)	C7—C2—C3—C4	−1.82 (18)
N2—C2—C3—C4	176.35 (12)	C7—C8—C9—C10	118.06 (14)
N2—C2—C7—C6	−174.59 (11)	C7—C8—C9—C14	−60.81 (16)
N2—C2—C7—C8	4.09 (17)	C8—N1—C1—N2	2.29 (18)
C1—N1—C8—C7	1.96 (16)	C8—N1—C1—C15	−176.27 (11)
C1—N1—C8—C9	−176.85 (11)	C8—C9—C10—C11	−177.93 (12)
C1—N2—C2—C3	−178.21 (12)	C8—C9—C14—C13	179.60 (12)
C1—N2—C2—C7	−0.05 (18)	C9—C10—C11—C12	−1.71 (19)
C2—N2—C1—N1	−3.24 (18)	C10—C9—C14—C13	0.71 (19)
C2—N2—C1—C15	175.27 (11)	C10—C11—C12—C13	0.8 (2)
C2—C3—C4—C5	−0.76 (19)	C11—C12—C13—C14	0.8 (2)
C2—C7—C8—N1	−4.93 (17)	C12—C13—C14—C9	−1.6 (2)
C2—C7—C8—C9	173.83 (11)	C14—C9—C10—C11	0.93 (19)
C3—C2—C7—C6	3.56 (17)		

### Hydrogen-bond geometry (Å, º)

**Table:**

*D*—H···*A*	*D*—H	H···*A*	*D*···*A*	*D*—H···*A*
C15—H15*A*···O1^i^	0.97	2.57	3.4199 (16)	146

Symmetry code: (i) −*x*+1, −*y*+1, −*z*+1.
